# Word pair classification during imagined speech using direct brain recordings

**DOI:** 10.1038/srep25803

**Published:** 2016-05-11

**Authors:** Stephanie Martin, Peter Brunner, Iñaki Iturrate, José del R. Millán, Gerwin Schalk, Robert T. Knight, Brian N. Pasley

**Affiliations:** 1 Defitech Chair in Brain-Machine Interface, Center for Neuroprosthetics, Ecole Polytechnique Fédérale de Lausanne, Switzerland; 2 Helen Wills Neuroscience Institute, University of California, Berkeley, CA, USA; 3 National Center for Adaptive Neurotechnologies, Wadsworth Center, New York State Department of Health, Albany, NY, USA; 4 Department of Neurology, Albany Medical College, Albany, NY, USA; 5 Department of Psychology, University of California, Berkeley, CA, USA

## Abstract

People that cannot communicate due to neurological disorders would benefit from an internal speech decoder. Here, we showed the ability to classify individual words during imagined speech from electrocorticographic signals. In a word imagery task, we used high gamma (70–150 Hz) time features with a support vector machine model to classify individual words from a pair of words. To account for temporal irregularities during speech production, we introduced a non-linear time alignment into the SVM kernel. Classification accuracy reached 88% in a two-class classification framework (50% chance level), and average classification accuracy across fifteen word-pairs was significant across five subjects (mean = 58%; p < 0.05). We also compared classification accuracy between imagined speech, overt speech and listening. As predicted, higher classification accuracy was obtained in the listening and overt speech conditions (mean = 89% and 86%, respectively; p < 0.0001), where speech stimuli were directly presented. The results provide evidence for a neural representation for imagined words in the temporal lobe, frontal lobe and sensorimotor cortex, consistent with previous findings in speech perception and production. These data represent a proof of concept study for basic decoding of speech imagery, and delineate a number of key challenges to usage of speech imagery neural representations for clinical applications.

Several neurological disorders limit verbal communication despite the patient being fully aware of what they want to say. These disorders include brainstem infarcts, traumatic brain injury, stroke and amyotrophic lateral sclerosis^1^. People with speech production impairments would benefit from a system that can infer intended speech directly from brain signals. Here, we used direct cortical recording (electrocorticography; ECoG) to examine if individual words could be selected during imagined speech within a binary classification framework.

One approach to decoding intended speech is to model the neural representation of speech imagery. Imagined speech (i.e., inner speech, silent speech, speech imagery, covert speech or verbal thoughts) is defined as the ability to generate internal auditory representations of speech sounds, in the absence of any external speech stimulation or self-generated overt speech. Despite intense investigation, the neural mechanisms underlying imagined speech remain poorly defined in part due to the lack of clear timing of inner speech, and the subjective nature of speech imagery. Functional magnetic resonance imaging studies have shown that imagined speech activates Wernicke’s area^2^,^3^,^4^,^5^,^6^,^7^,^8^ (superior temporal gyrus and superior temporal sulcus) and Broca’s area^9^,^10^ (inferior frontal gyrus) – two essential language areas involved in speech comprehension and production, respectively (see^11^,^12^ for reviews).

Although traditional brain imaging techniques have identified anatomical regions associated with imagined speech, these methods lack the temporal resolution to investigate the rapid temporal neural dynamics during imagined speech^13^. In contrast, electrocorticography is a direct neural recording method that allows monitoring brain activity with high spatial, temporal, and spectral resolution^14^. The high gamma band (HG; 70–150 Hz) in particular has been associated with both the spike rate and local field potentials of the underlying neural population^15^,^16^,^17^, and reliably tracks rapid neural fluctuations during speech perception and production^18^,^19^,^20^,^21^,^22^,^23^,^24^.

Previous studies were able to decode various stimulus features of imagined speech, such as vowels and consonants^19^,^25^, acoustic features^26^,^27^ and intended phonemes^28^,^29^. In this study, we took advantage of the high resolution offered by ECoG to evaluate the ability to identify individual words in a binary word classification task during imagined speech – using HG features in the time domain. However, speech production (both overt and imagined) is subject to temporal variations (speech onset delays and local stretching/compression) across repetitions of the same utterance^30^,^31^. As a result, a classifier that assumes fixed time features may not recognize two trials as belonging to the same class if the neural patterns were not temporally aligned. To overcome that limitation, we proposed a new classification framework that accounted for temporal variations during speech production (overt and imagined) by introducing time realignment in the feature map generation. For this, we used an imagined word repetition task cued with a word perception stimulus, and followed by an overt word repetition, and compared the results across the three conditions (listening, overt and imagined speech). As expected, high classification accuracy was obtained in the listening and overt speech condition where speech stimuli were directly observed. In the imagined speech condition, where speech is generated internally by the patient, results show for the first time that individual words in single trials were classified with statistically significant accuracy. The majority of electrodes carrying discriminative information were located in the superior temporal gyrus, inferior frontal gyrus and sensorimotor cortex – regions commonly associated with speech processing. Notably, the most robust decoding effects were observed in the temporal lobe electrodes.

## Results

### High gamma features

**Figure 1:**
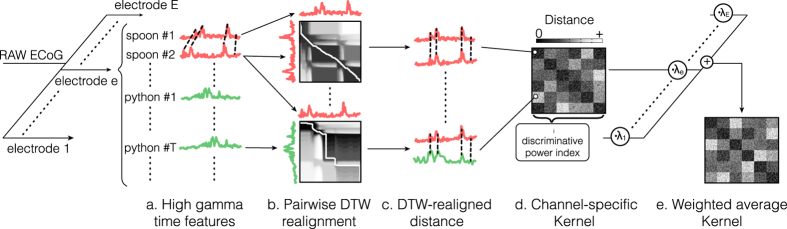
Experimental paradigm. Subjects were presented with an auditory stimulus that indicated one of six individual words (average length = 800 ms ± 20). Then, a cue appeared on the screen [describe what the cue is and where it appeared on the screen], and subjects had to imagined hearing the word they had just listened to. Finally, a second cue appeared, and subjects had to say the word out loud. Shaded areas represent the intervals extracted for classification. For both listening and overt speech condition, we extracted epochs from 100 ms before speech onset to 100 ms after speech offset. For the imagined speech condition, we extracted fixed length 1.5 sec epochs starting at cue onset, since there was no speech output.

Electrocorticographic (ECoG) recordings were obtained using subdural electrode arrays implanted in 5 patients undergoing neurosurgical procedures for epilepsy. Grid placement and duration of ECoG monitoring were based solely on the requirements of the clinical evaluation (Supplementary Fig. S1). We analyzed three conditions: word perception (listening condition), overt (speaking condition) and imagined word production (imagined condition) (Fig. 1). Each trial started with an auditory word stimulus presented through a loudspeaker (listening condition) – indicating one of 6 individual words (average length = 800 ms ± 20). Then, a visual cue appeared on the screen indicating to the patient to repeat the word silently (imagined condition). Finally, a second visual cue appeared and the patient had to repeat the word overtly (overt condition). By pacing the subject, the task was designed to minimize the behavioral variance in producing overt and imagined speech.

**Figure 2:**
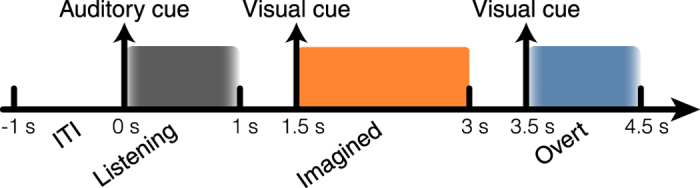
High gamma time course. (**a**) High gamma neural activity averaged across trials and z-scored with respect to the pre-auditory stimuli baseline condition (500 ms interval). The top-most plot displays the designed task, an example of averaged time course for a representative electrode and the averaged audio envelope (red line). (**b**) For the given electrodes and conditions (listening, imagined and overt speech), examples of individual trials (black) and their corresponding audio recording (red) for three different words (‘battlefield’, ‘swimming’ and ‘telephone’).

We analyzed z-scored high gamma time courses at different electrode locations, and compared the different conditions (listening, overt, and imagined speech). Word perception and production (both overt and imagined) evoked different high-gamma neural responses across many electrodes (Fig. 2a) in all participants (Supplementary Fig. S2). An exemplary electrode in the posterior superior temporal gyrus showed activation during all three conditions, while the neighboring electrode had activity only in the listening and overt speech conditions. An electrode in sensorimotor cortex showed sustained activity in the overt and imagined speech task. Finally, an electrode in the anterior temporal lobe, associated with speech production, exhibited activity only in the overt speech task, but not during listening or imagined speech. These results revealed the complex dynamics of speech perception and production (overt and imagined), and suggest that the neural representations underlying the different speech modalities are partially overlapping, yet dissociable^2^,^4^,^32^.

In the listening condition, the auditory stimuli were time-locked across repetitions (Fig. 2b; audio envelope in red; standard deviation of the onset delay averaged over all words = 0 ms). Alternatively, in the overt speech condition, temporal irregularities in the speech onset and word duration were observed across repetitions of the same utterance (Fig. 2b; standard deviation of the onset delay averaged over all words = 220 ms). Because high gamma neural activity is known to track the speech envelope^22^,^23^,^27^,^33^, we assumed that temporal variations in overt and imagined speech would also be represented by the measured neural responses. As such, a classifier that assumes fixed neural temporal features would not recognize two trials as belonging to the same class if the neural patterns are not aligned in time. To overcome this limitation, we applied a temporal realignment procedure in the feature map generation. The procedure was applied to both overt and imagined speech, as both conditions have been shown to be subject to similar speech production temporal variations^34^.

### Classification

We used support-vector machines^35^ (SVM) to perform pair-wise classification of different individual words in the three different speech conditions (overt, listening and imagined speech). We first extracted the high gamma using bandpass filtering in the 70–150 Hz range, extracted the envelope using the Hilbert transform. We then extracted epochs from 100 ms before speech onset to 100 ms after speech offset for both listening and overt speech condition. Average word length for both listening and overt speech conditions were 800 ms ± 20 and 766 ms ± 84, respectively. In the imagined speech condition, due to the lack of speech output, we extracted 1500 ms epochs starting at cue onset.

**Figure 3:**
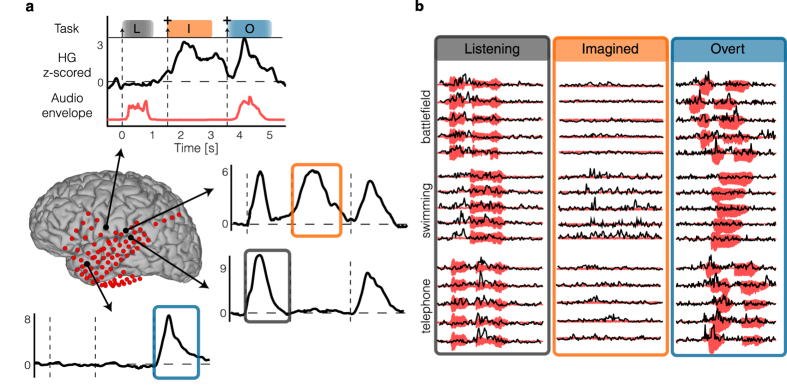
Neural time course alignment. (**a**) For each electrode separately, we extracted the high gamma time features. (**b**) We used dynamic time warping to realign the time series of each pair of trials, and (**c**) computed the DTW-distance between the pairwise realigned trials. (**d**) This gave rise to one similarity matrix per electrode (channel-specific kernel) that reflects how similar trial-pairs are after realignment. From the similarity matrix in d, we computed the discriminative power index (see Materials and methods for details). (**e**) The final kernel was computed as the weighted average of the individual kernels over all electrodes, based on their discriminative power index.

The Gaussian kernel is a widely used function used in SVM-classification. In this approach, the output of the classifier is based on a weighted linear combination of similarity measures (i.e., Euclidean distance) computed between a data point and each of the support vectors^35^. In our study, to deal with speech temporal irregularities, we incorporated time alignment in the kernel computation. We used dynamic time warping^30^,^36^ (DTW) to locally expand or compress two time series, and find their optimal alignment in time. Then, we computed the Euclidian distance between the realigned time series (DTW-distance), as the similarity measure for the kernel computation. As such, for each electrode separately, we computed the DTW-distance between each pair of trials (Fig. 3a–c). This led to one kernel matrix per electrode (Fig. 3d). To build the final kernel function, we computed the weighted average of the kernel matrices over all electrodes (Fig. 3e; multiple kernel learning^37^). The weighting was based on the discriminative power index of each individual electrode, which quantified the difference between the “within” class versus “between” class distances distribution (see Materials and methods for details).

**Figure 4:**
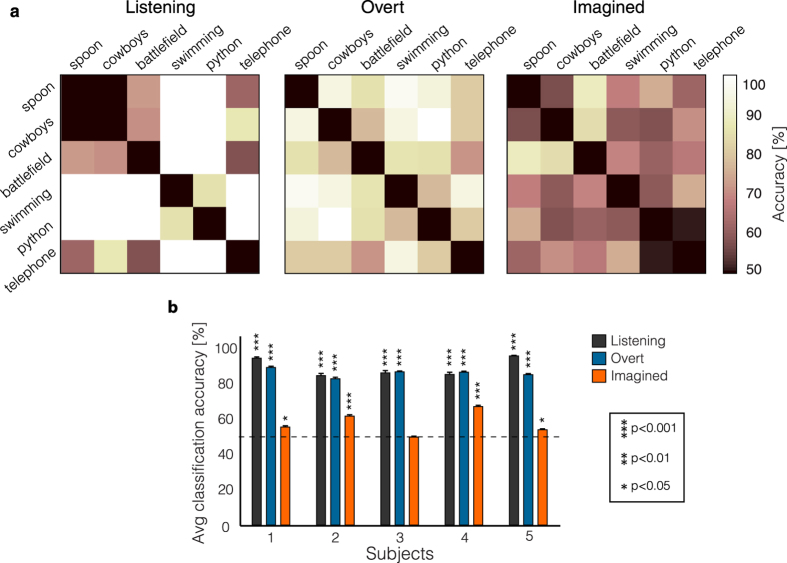
Classification accuracy. (**a**) Pairwise classification accuracy in the testing set for the listening (left panel), overt speech (middle panel) and imagined speech condition (right panel) for a subject with good temporal coverage (S4). (**b**) Average classification accuracy across all pairs of words for each subject and condition (listening, overt and imagined speech). Error bars denote SEM.

In the imagined speech condition, pairwise classification accuracy reached 88.3% for one classification pair in a subject with extensive left temporal coverage (subject 4; Fig. 4a). Eight out of fifteen word-pairs were classified significantly higher than chance level (p < 0.05; randomization test; FDR correction), exceeding the number of pairs expected by chance (0.05 × 15 = 0.75). As expected, higher classification accuracy was obtained in the listening and overt speech conditions where speech stimuli were directly observed. For both conditions, pairwise classification accuracy approached 100% in some comparisons, and twelve and fifteen out of fifteen pairs were significantly above chance, respectively (p < 0.05; randomization test; FDR correction).

Classification accuracy varied across subjects and pairs of words. In 4 out of 5 subjects, classification accuracy over all word pairs was significant in the imagined speech condition (Fig. 4b; p < 0.05; one-sample t-test; FDR correction), while the last subject was not significantly better than chance level (mean = 49.8%; p > 0.5; one-sample t-test; FDR correction). For listening and overt speech conditions, classification accuracy over all word pairs was again significant in all four subjects, and ranged between 83.0% and 96.0% (p < 10^−4^; one-sample t-test; FDR correction).

At the population level, average classification accuracy across all pairs was above chance level in all three conditions (Fig. 4b; listening: mean = 89.4% p < 10^−4^; overt speech: mean = 86.2%, p < 10^−5^; imagined speech: mean = 57.7%; p < 0.05; one-sample t-tests; FDR correction). A repeated measure 1-way ANOVA with experimental condition as a factor confirmed a difference among conditions (F_(2,12)_ = 56.3, p < 10^−5^). Post-hoc t-tests showed that the mean classification accuracy for listening was not significantly different from the overt speech (p > 0.1; two-sample t-test; FDR correction). Both were significantly higher than the imagined speech classification accuracy (p < 0.005; two-sample t-test; FDR correction). Although the classification accuracy for imagined speech was lower than for listening and overt speech, the imagery classification results provide evidence that high gamma time course during imagined speech contained information to distinguish pairs of words.

To assess the impact of the neural activity realignment procedure in classification accuracy, we evaluated the improvement of DTW alignment compared to when no alignment was applied. The results showed that for both the overt and imagined speech conditions, the average classification accuracy was y reduced when no alignment was applied (Supplementary Fig. S3; p < 0.05; two-sample t-test; FDR correction). On the other hand, for the listening condition – in which trials were time-locked to stimulus onset – the DTW procedure did not improve the classification accuracy (p > 0.5; two-sample t-test; FDR correction).

The inability to directly measure temporal variability in the imagery condition remains a major limiting factor for classification accuracy, despite the realignment procedure we employed. In the imagined speech condition, due to the lack of speech output, we could only extract trials at cue onset rather than at the true onset of speech imagery. To investigate the impact of this limitation on classification accuracy, we analyzed data from the overt speech condition where the auditory stimulus is directly measured. The results showed that classification accuracy in the overt speech condition was reduced when extracting epochs at cue onset, compared to when epochs were extracted between speech onset and offset (Supplementary Fig. S3; p < 0.05; two-sample t-test). This further highlights limitations in the realignment algorithm, and indicates that imagery classification accuracy may be increased by developing enhanced methods to define imagined speech onset and offset.

### Anatomical distribution of discriminant electrodes

To assess how the brain areas important for word classification vary across experimental conditions, we analyzed the anatomical distribution of the electrodes carrying discriminative information in the three different conditions. For each electrode and condition, we computed a discriminative power index that reflected the predictive power of each electrode in the classification process (see Materials and methods for details).

**Figure 5:**
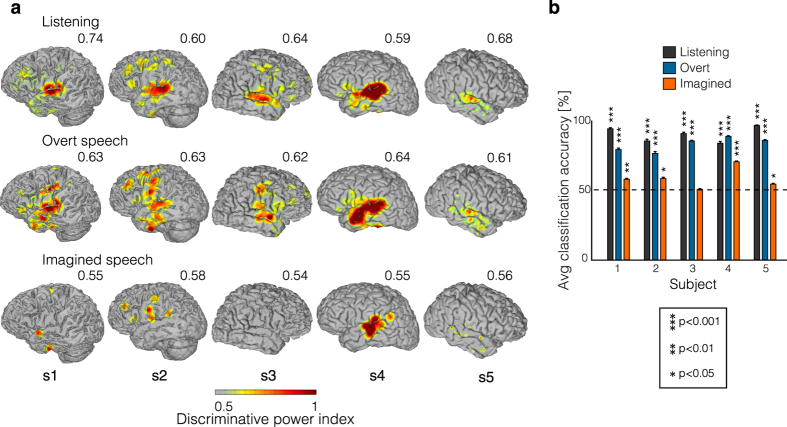
Discriminative information. (**a**) Discriminative power measured as the areas under the ROC curve (thresholded at p < 0.05; uncorrected; see Materials and methods for details), and plotted on each individual’s brain. Each is scaled to the maximum absolute value of discriminative power index (indicated by the number above each cortical map). (**b**) Average classification accuracy across all pairs of words for each subject using only temporal electrodes for the listening (top panel), overt speech (middle panel) and imagined speech (bottom panel). Error bars denote SEM.

Figure 5a shows the anatomical distribution of the discriminative power index across each condition (heat map thresholded at p < 0.05; uncorrected). Overall, the highest discriminative information was located in the temporal gyrus, inferior frontal gyrus and sensorimotor gyrus – regions commonly associated with speech processing. Anatomical differences between conditions were assessed for significant electrodes (188 electrodes significant in at least one condition; p < 0.05; FDR correction), using an unbalanced Two-Way ANOVA with interactions, with experimental condition (listening, overt and imagined speech) and anatomical region (superior temporal gyrus (STG), inferior frontal gyrus (IFG) and sensorimotor cortex (SMC)) as factors. The main effect of experimental condition was significant [F_(2, 555)_ = 29.1, p < 10^−15^], indicating that the discriminative information in the classification process was different across conditions. Post-hoc t-tests with Bonferroni correction showed that the overall discriminative power was higher in the listening (mean = 0.56) and overt speech condition (mean = 0.56) than in the imagined speech (mean = 0.53; p < 10^−10^; unpaired two-sample t-test; Bonferroni correction), at the level of single electrodes. The main effect of anatomical region was also significant [F_(2, 555)_ = 7.18, p < 0.001]. Post-hoc t-tests indicated stronger discriminative information in the STG (mean = 0.55) than in the inferior frontal gyrus (mean = 0.54; p < 0.05; unpaired two-sample t-test; Bonferroni correction), but not than the SMC (mean = 0.54; p > 0.05; unpaired two-sample t-test; Bonferroni correction). The interaction between gyrus and experimental condition was also significant [F_(4, 555)_ = 6.7; p < 10^−4^]. Specifically, The discriminative power in the STG was higher for listening (mean = 0.57) and overt speech (mean = 0.56) than for imagined speech (mean = 0.53; p < 10^−10^; unpaired two-sample t-test; Bonferroni correction). In addition, the discriminative power in the sensorimotor cortex was higher in the overt condition (mean = 0.57), than in the listening (mean = 0.54) and imagined condition (mean = 0.53; p < 0.001; unpaired two-sample t-test; Bonferroni correction). Similarly, the frontal electrodes provided more discriminative information in the overt speech (mean = 0.55) than in the imagined speech condition (mean = 0.53; p < 10^−4^; unpaired two-sample t-test; Bonferroni correction). Post-hoc t-tests also showed that the discriminative power in the listening condition was higher in the STG (mean = 0.56) than in the IFG (mean = 0.54) and SMC (mean = 0.54; p < 0.05; unpaired two-sample t-test; FDR correction). Finally, no significant differences across gyri were observed in the imagined speech condition (p > 0.5; unpaired two-sample t-test; Bonferroni correction).

While the anatomic locations (i.e. STG, IFG and SMC) that give rise to the best word discrimination in the listening and overt speech conditions were consistent across subjects, discriminative anatomic locations in the imagined condition varied. To further investigate brain areas and based on a number of previous studies demonstrating its role in auditory imagery^2^,^3^,^4^,^5^,^38^, we performed the classification using only electrodes from the superior temporal gyrus (Fig. 5b). In the imagery condition, classification accuracy using STG electrodes was significant in four out of five subjects (p < 0.05; one-sample t-test; FDR correction), while it was not significant in S3 (p > 0.5; one-sample t-test; FDR correction). At the group level, classification using only temporal electrodes was significant (mean = 58.0%; p < 0.05; one-sample t-test; FDR correction). For both, listening and overt speech conditions, classification accuracy was significant in all individual subjects when using only STG electrodes (p < 10^−4^; one-sample t-test; FDR correction), as well as at the group level (mean listening = 89.5% and mean overt speech = 82.6%; p < 10^−4^; one-sample t-test; FDR correction). This provides preliminary evidence that superior temporal gyrus alone could drive auditory imagery decoding, but that other areas such as frontal cortex and sensorimotor cortex could also contribute.

## Discussion

Our results provide the first demonstration of single-trial neural decoding of words during imagined speech production. We developed a new binary classification approach that accounted for temporal variations in the high gamma neural activity across speech utterances. We used support-vector machines to classify individual words in a word pair, and introduced a non-linear time alignment into the kernel to deal with internal speech production variability. At the group level, average classification accuracy across all pairs was significant in all three conditions. Two subjects that exhibited the lowest classification scores had right hemisphere coverage and were right handed typically associated with left hemisphere language dominance^39^. This could contribute to differences in accuracy across subjects. However, more data are required to delineate the effect of hemisphere coverage in the decoding process. The anatomic locations that led to the best word discrimination in the listening and overt speech conditions were consistent across subjects. All three anatomical regions (STG, IFG and SMC) provided information in the classification process. In the imagery condition, anatomical areas with the highest predictive power were more variable across subjects. The results revealed that the STG alone could drive auditory imagery decoding, but that other areas, such as the IFG and SMC also contribute.

An important component of the study is the application of dynamic time warping in the classification framework to account for speech production temporal irregularities. This technique maximizes alignment of the neural activity time courses without knowledge of the exact onset of the events. This approach proved useful for studying imagined speech where no behavior or stimuli are explicitly observed. In contrast, DTW did not improve accuracy in the listening condition, where neural activity is already time-locked to stimulus events. This highlights the usefulness of a time alignment procedure such as using DTW for modeling the neural activity of unobserved behavioral events such as imagery. We also note the limitations of DTW in noisy environments suggesting that imagery results may be improved by developing more robust realignment techniques. We also show that overt speech classification accuracy was improved when epochs were selected from speech onset/offset, as compared to when they were extracted from cue onset. This suggests that the results may be improved by developing enhanced methods to define imagined speech onset and offset. Ideas for possible future directions would be to improve experimental paradigms (i.e. button press, karaoke-task, etc.), define improved behavioral or neural metrics that correlates with speech onset/offset and increased training in imagery prior to ECOG recording.

Despite intense investigation, it is still unclear how the content of imagined speech is processed in the human cortex. Different tasks – such as word repetition, letter or object naming, verb generation, reading, rhyme judgment, counting – involve different speech production processes, ranging from lexical retrieval to phonological or even phonetic encoding^12^. In this study, we chose the set of auditory stimuli to maximize variability in several speech feature spaces (acoustic features, number of syllable, semantic categories), but to minimize word length variance. Our approach does not allow us to investigate which specific speech features provided information and allowed classification; i.e., if the discrimination was based on acoustic, phonetic, phonological, semantic or abstract features within speech perception, comprehension or production. Given that several brain areas were involved, it is likely that various features of speech were involved in the classification process.

Several additional limitations precluded high word prediction accuracy during imagined speech. First, we were limited by the electrode location and duration of implantation that was not designed for the experiments, but solely for clinical needs. Higher density grids placed at a specific locations in the posterior superior temporal gyrus, frontal cortex and/or sensorimotor cortex that are active during imagined speech would provide higher spatial resolution and potentially enhanced discriminating signals^40^. Further, subjects were not familiarized with the task beforehand (i.e. no training), and due to time constraints in the epilepsy-monitoring unit, we were unable to monitor subjects’ performance or vividness during speech imagery. We also could not reject pronunciation and grammatical mistakes, as we did in the overt speech condition. We propose it would be beneficial to train subjects on speech imagery prior to surgery to enhance task performance.

Finally, although our study is a proof of concepts for basic decoding of speech imagery, many issues still need to be tackled to prove the feasibility for a clinical application. Our current approach was limited in the set of choices available, and only tests binary classification between word pairs. In addition, the effect size is small, and likely not clinically significant for a communication interface. Classification of individual words among multiple other words or continuous speech decoding would be a more realistic clinical scenario. An alternative would be classifying phonemes, which forms the building blocks of speech instances. Decoding vowels and consonants in overt and imagined words using electrocorticographic signals in humans has shown promising results^28^,^29^, and would allow generating a larger lexicon from a fewer number of classes (60–80 phonemes in spoken English^31^).

## Materials and Methods

### Subjects and data acquisition

Electrocorticographic (ECoG) recordings were obtained using subdural electrode arrays implanted in 5 patients undergoing neurosurgical procedures for epilepsy. All patients volunteered and gave their informed consent (experimental protocol was approved by the Albany Medical College Institutional Review Board and methods were carried out in accordance with the approved guidelines and regulations) before testing. The implanted electrode grids (Ad-Tech Medical Corp., Racine, WI; PMT Corporation, Chanhassen, MN) consisted of platinum–iridium electrodes (4 mm in diameter, 2.3 mm exposed) that were embedded in silicon and spaced at an inter-electrode distance of 4–10 mm. Grid placement and duration of ECoG monitoring were based solely on the requirements of the clinical evaluation (Supplementary Fig. S1).

ECoG signals were recorded at the bedside using seven 16-channel g.USBamp biosignal acquisition devices (g.tec, Graz, Austria) at a sampling rate of 9,600 Hz. Electrode contacts distant from epileptic foci and areas of interest were used for reference and ground. Data acquisition and synchronization with the task presentation were accomplished using BCI2000 software^41^,^42^. All electrodes were subsequently downsampled to 1,000 Hz, corrected for DC shifts, and band pass filtered from 0.5 to 200 Hz. Notch filters at 60 Hz, 120 Hz and 180 Hz were used to remove electromagnetic noise. The time series were then visually inspected to remove the intervals containing ictal activity as well as electrodes that had excessive noise (including broadband electromagnetic noise from hospital equipment or poor contact with the cortical surface). Finally, electrodes were re-referenced to a common average. Imagined speech trials were carefully analyzed to remove those that were contaminated by overt speech. Overt speech trials that had grammar mistakes were also removed.

In addition to the ECoG signals, we acquired the subject’s voice through a dynamic microphone (Samson R21s) that was rated for voice recordings (bandwidth 80–12,000 Hz, sensitivity 2.24 mV/Pa) and placed within 10 cm of the patient’s face. We used a dedicated 16-channel g.USBamp to amplify and digitize the microphone signal in sync with the ECoG data. Finally, we verified the patient’s compliance in the imagined task using an eye-tracker (Tobii T60, Tobii Sweden).

### Experimental paradigm

We used a word repetition task (overt and imagined) cued with an auditory stimulus presentation. Each trial started with an auditory cue presented through a loudspeaker indicating one of six individual words (average length = 800 ms ± 20) to repeat; 800 ms after the end of the auditory stimulus a cross was displayed on the screen for 1500 ms (Fig. 1). This indicated to the subjects to imagine hearing the word again in their mind. Subjects were instructed to “imagine hearing”, because we were interested in the auditory perceptual representation induced by imagery, rather than kinesthetic (imagine saying words) or visual (imagine seeing words) representations. Finally, after 500 ms of blank screen, a second cross was displayed for 1500 ms, and subjects had to repeat the word out loud. The choice of stimuli was carefully chosen to maximize variability in terms of acoustic features, number of syllables and semantic categories, but minimize word length variability (variance in word length 20 ms; ‘spoon’, ‘cowboy’, ‘battlefield’, ‘swimming’, ‘python’, ‘telephone’). Trials were repeated randomly between 18 and 24 times. The precise task design and timing is summarized in Fig. 1. The microphone recording was used to verify that subjects were not producing audible speech during imagery, as well as monitoring the behavior (speech onset and word length) during overt speech. For each condition, we analyzed high gamma activity (HG) and built separate, independent classifiers, which allowed us to compare classification accuracy and discriminative information across perception and imagery tasks.

### Feature extraction

To generate input features for the classifier, we filtered the ECoG signal in the high gamma (HG) frequency band (70–150 Hz; hamming window non-causal filter of order 20), and extracted the envelope using the Hilbert transform. Prior to model fitting, we downsampled the HG signal to 100 Hz to reduce computational load. For both listening and overt speech condition, we extracted epochs from 100 ms before speech onset to 100 ms after speech offset (unless otherwise stated). For the imagined speech condition, because there was no speech output we extracted fixed 1.5 s epochs starting at cue onset.

### Classification

To classify the different pairs of words, we used support-vector machines^35^ (SVM). This classifier maps the original input features into a higher dimensionality non-linear feature space via a kernel function. Its main advantages are robustness to overfitting (due to the inclusion of a regularization term) and underfitting^43^ (due to the higher-dimensional mapping of the features). The general approach of SVM is described as follows:


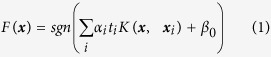


where 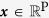, with P the number of features. *K* is the kernel function that transforms the input data ***x*** into a non-linear feature map. *α*_*i*_ ∈ [0, *C*] are weights for the support vectors. The constant C is the soft margin parameter, and controls the trade-off between classification error on the training set and smoothness of the decision boundary. *t*_*i*_ is the label of sample i, and *β*_*0*_ is the offset of the separating hyperplane from the origin. For all the computations, we used the LIBSVM package^44^.

### SVM-Kernel computation

Speech production (overt and imagined) is subject to temporal variability (speech onset delays and local stretching/compression) across repetitions of the same instance. A classifier that assumes fixed time features might not recognize two trials as belonging to the same class if the neural patterns are not aligned in time. In order to deal with speech temporal irregularities, we developed a classification approach that incorporated non-linear time alignment in the kernel computation, using dynamic time warping^30^,^36^ (DTW; see section “Dynamic time warping” for details). The use of DTW-distances as an SVM-kernel function has shown its superiority over hidden Markov models for speech recognition. DTW provides a distance between two realigned time series that reflects how similar both are when maximally aligned (Fig. 3b,c). For each electrode separately, we computed the DTW-distance between each pair of trials (Fig. 3a–c). This gave rise to one kernel matrix per electrode (Fig. 3d). For the final kernel computation, we used a multiple kernel learning approach^37^,^45^ (MKL; see section “Multiple kernel learning” for details) to deal with the multiple kernel matrices – by doing a weighted average of the kernels associated with each electrode (Fig. 3e). The weighting was based on the discriminative power index of each individual electrode, which quantified the difference between the “within” class versus “between” class distances distribution (see section “Discriminative power index” for details).

### Dynamic time warping

The main idea behind DTW is to locally stretch or compress (i.e., warp) two time series 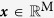 and 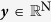 where M and N are the number of time samples in 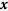 and 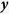 respectively^36^. For each electrode separately, we computed the DTW for each pair of trials as follows:

Let 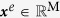 and 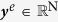, be the temporal features associated with two trials from electrode e. Each trial corresponded to a single word in one condition, represented as its associated HG features (see previous subsection). Trials had different length for both listening and overt speech conditions (M ≠ N), but equal length for the imagined speech condition (M = N). First, a pattern matching matrix 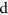 was computed between each time point pairs (Fig. 3b), as follows:


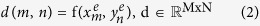


where *d*(*m, n*) is the pattern matching index between 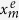 and 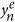 at the time sample m and n, respectively, and 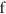 an arbitrary distance metric. In this study, we used the Euclidean distance defined as 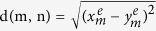. Given a warping path 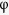, the average accumulated distortion between both warped signals is defined by:


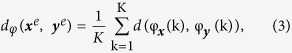


where 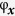 and 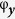 are the warping functions of length K (that remap the time indices of 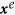 and 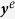, respectively). The optimal warping path 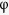 (white line in Fig. 3b), chooses the indices of 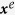 and 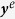 in order to minimize the overall accumulated distance:


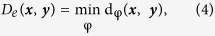


where 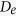 is the optimal realigned Euclidean distance between ***x*** and ***y*** at a given electrode e. A dynamic programming approach was used to solve the global distance efficiently^46^.

### Multiple kernel learning

Once the realigned DTW distances of each electrode were computed, we built the kernel for the SVM classification by summing the weighted DTW kernels (fixed-rule multiple kernel^37^), as follows:


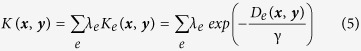


where 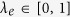 is the normalized discriminative power index of electrode e, 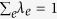; and 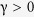 a free parameter.

### Discriminative power index ( 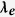 ) computation

Among the many ways to compute discriminative power between classes, we opted for the area under the receiver operating characteristic curve^35^ (ROC), which measured the performance of a linear discriminant trained on the features given by the kernel 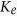. This index reflected the difference between the “within” class versus “between” class distances. Entries of the distance matrices representing the within class distance had label = 0 and entries of the distance matrix representing the between class distance had label = 1. For each electrode, the discriminative power index was calculated from the data with 0/1 labels and the corresponding realigned DTW distance matrices.

### Classifier training and performance evaluation

Due to the limited number of trials, the classification performance was evaluated using a leave-one-out cross validation, where the test set was composed of one sample per class for each fold. With the training data, an inner leave-one-out cross validation was performed to find the optimal free parameters 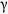 and 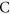 using a grid search approach, and were then fixed for the test evaluation. We also computed the discriminative power index 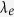 on the training set. In the paper, we reported the classification accuracy as the percentage of correctly classified trials averaged on the outer loop cross-validation.

### Statistics

For each condition, we evaluated statistical significance for each pair of words using randomization tests. After extracting the high gamma time features, we randomly shuffled 1,000 times the trial labels, and applied the exact same approach as in the actual classification process; we extracted HG time features, split into training and testing set, applied DTW at the trial level, built the kernel function, performed grid search on the inner loop, built the final model and evaluated the accuracy on the outer loop testing set. The averaged accuracy across cross-validated testing set yielded one value in the null distribution. The proportion of shuffled classification accuracy values greater than the observed accuracy yielded the p-value that the observed accuracy was due to chance. We corrected for multiple comparison using False Discovery Rate^47^ (FDR). The average of the null distributions was not significantly different from the expected value of chance level (50%; p > 0.5; one-sample t-test). For each individual subject, we evaluated the significance level of the classification accuracy across all word pairs using one-sample t-tests against chance level (50%). Here again, we corrected for multiple comparison using FDR. Finally, we evaluated the significance level of the average classification accuracy across subjects using one-sample t-test again chance level. To investigate possible anatomical differences between conditions, all electrodes carrying significant discriminative information in at least one condition (listening, overt or imagined; p < 0.05; Bonferroni correction) were selected for an unbalanced Two-Way ANOVA with interactions, with experimental condition (listening, overt and imagined) and anatomical region (superior temporal gyrus, inferior frontal gyrus and sensorimotor cortex) as factors. Prior to ANOVA, we performed Mauchly’s test to ensure the sphericity of data^48^. To define the significance level of discriminative power of each single electrode, we computed the discriminative power indices for the above-mentioned shuffled data. The discriminative power index yielded one value in the null distribution. For each electrode, the proportion of shuffled index values greater than the observed value yielded the p-value that the observed discriminative power of the electrode was due to chance.

## Additional Information

**How to cite this article**: Martin, S. *et al*. Word pair classification during imagined speech using direct brain recordings. *Sci. Rep.* **6**, 25803; doi: 10.1038/srep25803 (2016).

## Supplementary Material

Supplementary Information

## References

[b1] Smith E. Locked-in syndrome. BMJ 330, 406–409 (2005). 15718541 10.1136/bmj.330.7488.406 PMC549115

[b2] Yetkin F. Z. . A comparison of functional MR activation patterns during silent and audible language tasks. AJNR Am. J. Neuroradiol. 16, 1087–1092 (1995). PMC8337795 7639132

[b3] McGuire P. K. . Functional anatomy of inner speech and auditory verbal imagery. Psychol. Med. 26, 29–38 (1996). 8643761 10.1017/s0033291700033699

[b4] Palmer E. D. . An Event-Related fMRI Study of Overt and Covert Word Stem Completion. NeuroImage 14, 182–193 (2001). 11525327 10.1006/nimg.2001.0779

[b5] Shergill S. S. . A functional study of auditory verbal imagery. Psychol. Med. 31, 241–253 (2001). 11232912 10.1017/s003329170100335x

[b6] Aleman A. The Functional Neuroanatomy of Metrical Stress Evaluation of Perceived and Imagined Spoken Words. Cereb. Cortex 15, 221–228 (2004). 10.1093/cercor/bhh124 15269107

[b7] Aziz-Zadeh L., Cattaneo L., Rochat M. & Rizzolatti G. Covert speech arrest induced by rTMS over both motor and nonmotor left hemisphere frontal sites. J. Cogn. Neurosci. 17, 928–938 (2005). 15969910 10.1162/0898929054021157

[b8] Geva Correia & Warburton. Diffusion tensor imaging in the study of language and aphasia. Aphasiology 25, 543–558 (2011).

[b9] Hinke R. M. . Functional magnetic resonance imaging of Broca’s area during internal speech. Neuroreport 4, 675–678 (1993). 10.1097/00001756-199306000-00018 8347806

[b10] Huang J., Carr T. H. & Cao Y. Comparing cortical activations for silent and overt speech using event-related fMRI. Hum. Brain Mapp. 15, 39–53 (2002). 10.1002/hbm.1060 PMC6871953 11747099

[b11] Price C. J. A review and synthesis of the first 20 years of PET and fMRI studies of heard speech, spoken language and reading. NeuroImage 62, 816–847 (2012). 10.1016/j.neuroimage.2012.04.062 PMC3398395 22584224

[b12] Perrone-Bertolotti M., Rapin L., Lachaux J.-P., Baciu M. & Lœvenbruck H. What is that little voice inside my head? Inner speech phenomenology, its role in cognitive performance, and its relation to self-monitoring. Behav. Brain Res. 261, 220–239 (2014). 10.1016/j.bbr.2013.12.034 24412278

[b13] Towle V. L. . ECoG gamma activity during a language task: differentiating expressive and receptive speech areas. Brain 131, 2013–2027 (2008). 10.1093/brain/awn147 PMC2724904 18669510

[b14] Ritaccio A. . Proceedings of the Fifth International Workshop on Advances in Electrocorticography. Epilepsy Behav. 41, 183–192 (2014). 10.1016/j.yebeh.2014.09.015 PMC4268064 25461213

[b15] Miller K. J. . Spectral changes in cortical surface potentials during motor movement. J. Neurosci. Off. J. Soc. Neurosci. 27, 2424–2432 (2007). 10.1523/JNEUROSCI.3886-06.2007 PMC6673496 17329441

[b16] Boonstra T. W., Houweling S. & Muskulus M. Does Asynchronous Neuronal Activity Average out on a Macroscopic Scale? J. Neurosci. 29, 8871–8874 (2009). 10.1523/JNEUROSCI.2020-09.2009 PMC6665427 19605624

[b17] Lachaux J.-P., Axmacher N., Mormann F., Halgren E. & Crone N. E. High-frequency neural activity and human cognition: past, present and possible future of intracranial EEG research. Prog. Neurobiol. 98, 279–301 (2012). 10.1016/j.pneurobio.2012.06.008 PMC3980670 22750156

[b18] Crone N. E., Boatman D., Gordon B. & Hao L. Induced electrocorticographic gamma activity during auditory perception. Brazier Award-winning article, 2001. Clin. Neurophysiol. Off. J. Int. Fed. Clin. Neurophysiol. 112, 565–582 (2001). 10.1016/s1388-2457(00)00545-9 11275528

[b19] Pei X. . Spatiotemporal dynamics of electrocorticographic high gamma activity during overt and covert word repetition. NeuroImage 54, 2960–2972 (2011). 10.1016/j.neuroimage.2010.10.029 PMC3020260 21029784

[b20] Flinker A., Chang E. F., Barbaro N. M., Berger M. S. & Knight R. T. Sub-centimeter language organization in the human temporal lobe. Brain Lang. 117, 103–109 (2011). 10.1016/j.bandl.2010.09.009 PMC3025271 20961611

[b21] Llorens A., Trébuchon A., Liégeois-Chauvel C. & Alario F.-X. Intra-Cranial Recordings of Brain Activity During Language Production. Front. Psychol. doi: 10.3389/fpsyg.2011.00375 (2011). PMC3246222 22207857

[b22] Pasley B. N. . Reconstructing Speech from Human Auditory Cortex. Plos Biol. 10, e1001251 (2012). 10.1371/journal.pbio.1001251 PMC3269422 22303281

[b23] Kubanek J., Brunner P., Gunduz A., Poeppel D. & Schalk G. The Tracking of Speech Envelope in the Human Cortex. Plos ONE 8, e53398 (2013). 10.1371/journal.pone.0053398 PMC3542338 23408924

[b24] Hermes D. . Cortical theta wanes for language. NeuroImage 85, 738–748 (2014). 10.1016/j.neuroimage.2013.07.029 23891904

[b25] Ikeda S. . Neural decoding of single vowels during covert articulation using electrocorticography. Front. Hum. Neurosci. 125, doi: 10.3389/fnhum.2014.00125 (2014). PMC3945950 24639642

[b26] Guenther F. H. . A Wireless Brain-Machine Interface for Real-Time Speech Synthesis. Plos ONE 4, e8218 (2009). 10.1371/journal.pone.0008218 PMC2784218 20011034

[b27] Martin S. . Decoding spectrotemporal features of overt and covert speech from the human cortex. Front. Neuroengineering, doi: 10.3389/fneng.2014.00014 (2014). PMC4034498 24904404

[b28] Brumberg J. S., Wright E. J., Andreasen D. S., Guenther F. H. & Kennedy P. R. Classification of intended phoneme production from chronic intracortical microelectrode recordings in speech-motor cortex. Front. Neurosci. doi: 10.3389/fnins.2011.00065 (2011). PMC3096823 21629876

[b29] Herff C. . Brain-to-text: decoding spoken phrases from phone representations in the brain. Front. Neurosci. doi: 10.3389/fnins.2015.00217 (2015). PMC4464168 26124702

[b30] Rabiner L. R. Fundamentals of speech recognition. (PTR Prentice Hall, 1993).

[b31] Vaseghi S. V. Multimedia signal processing: theory and applications in speech, music and communications. (J. Wiley, 2007).

[b32] Rosen H. J., Ojemann J. G., Ollinger J. M. & Petersen S. E. Comparison of Brain Activation during Word Retrieval Done Silently and Aloud Using fMRI. Brain Cogn. 42, 201–217 (2000). 10.1006/brcg.1999.1100 10744920

[b33] Mesgarani & Chang E. F. Selective cortical representation of attended speaker in multi-talker speech perception. Nature 485, 233–236 (2012). 10.1038/nature11020 PMC3870007 22522927

[b34] Hubbard T. L. Auditory imagery: Empirical findings. Psychol. Bull. 136, 302–329 (2010). 10.1037/a0018436 20192565

[b35] Hastie T. The elements of statistical learning: data mining, inference, and prediction. (Springer, 2009).

[b36] Sakoe H. & Chiba S. Dynamic programming algorithm optimization for spoken word recognition. IEEE Trans. Acoust. Speech Signal Process. 26, 43–49 (1978).

[b37] Gönen M. & Ethem A. Multiple kernel learning algorithms. Journal of machine learning research 2211–2268 (2011).

[b38] Pei X., Barbour D. L., Leuthardt E. C. & Schalk G. Decoding vowels and consonants in spoken and imagined words using electrocorticographic signals in humans. J. Neural Eng. doi: 10.1088/1741-2560/8/4/046028 (2011). PMC3772685 21750369

[b39] Toga A. W. & Thompson P. M. Mapping brain asymmetry. Nat. Rev. Neurosci. 4, 37–48 (2003). 10.1038/nrn1009 12511860

[b40] Wodlinger B., Degenhart A. D., Collinger J. L., Tyler-Kabara E. C. & Wei Wang. The impact of electrode characteristics on electrocorticography (ECoG). In 3083–3086. doi: 10.1109/IEMBS.2011.6090842 (IEEE, 2011). 22254991

[b41] Schalk G., McFarland D. J., Hinterberger T., Birbaumer N. & Wolpaw J. R. BCI2000: A General-Purpose Brain-Computer Interface (BCI) System. IEEE Trans. Biomed. Eng. 51, 1034–1043 (2004). 10.1109/TBME.2004.827072 15188875

[b42] Schalk G. A practical guide to brain-computer interfacing with BCI2000: general-purpose software for brain-computer interface research, data acquisition, stimulus presentation, and brain monitoring. (Springer, 2010).

[b43] Stanikov A., Aliferis C. F., Hardin D. P. & Guyon I. In A Gentle Introduction to Support Vector Machines in Biomedicine, Volume 1: Theory and Methods (Singapore: World Scientific Publishing Co. Pte. Ltd., 2011).

[b44] Chang C.-C. & Lin C.-J. LIBSVM: A library for support vector machines. ACM Trans. Intell. Syst. Technol. 2, 1–27. Available at : http://www.csie.ntu.edu.tw/~cjlin/libsvm (Date of access: 1/11/2014) (2011).

[b45] Shimodaira H., Nom K., Nakai M. & Sagayama S. Dynamic Time-Alignment Kernel in Support Vector Machine. In 921–928 (2001).

[b46] Ellis D. *Dynamic time warping (DTW) in Matlab*. Available at: http://www.ee.columbia.edu/~dpwe/resources/matlab/dtw/ (Date of access: 11/12/2013) (2003).

[b47] Benjamini Y. & Hochberg Y. Controlling the False Discovery Rate: A Practical and Powerful Approach to Multiple Testing. J. R. Stat. Soc. Ser. B Methodol. 57, 289–300 (1995).

[b48] Mauchly J. W. Significance Test for Sphericity of a Normal n-Variate Distribution. Ann. Math. Stat. 11, 204–209 (1940).

