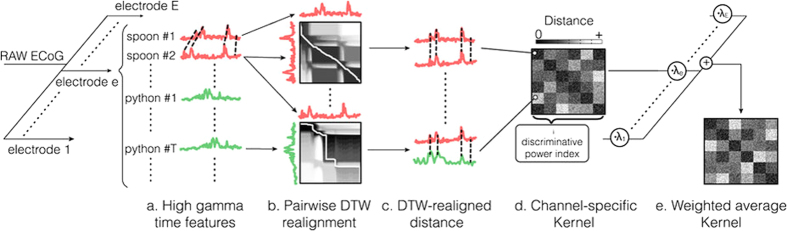# Corrigendum: Word pair classification during imagined speech using direct brain recordings

**DOI:** 10.1038/srep44509

**Published:** 2017-03-23

**Authors:** Stephanie Martin, Peter Brunner, Iñaki Iturrate, José del R. Millán, Gerwin Schalk, Robert T. Knight, Brian N. Pasley

Scientific Reports 6: Article number: 2580310.1038/srep25803; published online: 05 11 2016; updated: 03 23 2017

This Article contains errors in the order of Figures 1, 2 and 3. Figure 1 was published as Figure 2, Figure 2 was published as Figure 3 and Figure 3 was published as Figure 1. The correct Figures appear below. The legends for the Figures are correct.

## Figures and Tables

**Figure 1:**
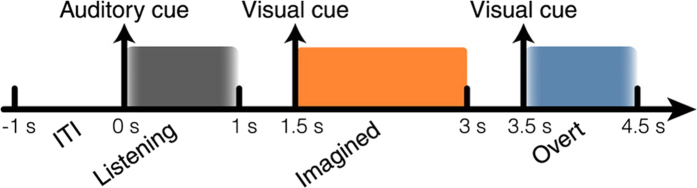


**Figure 2:**
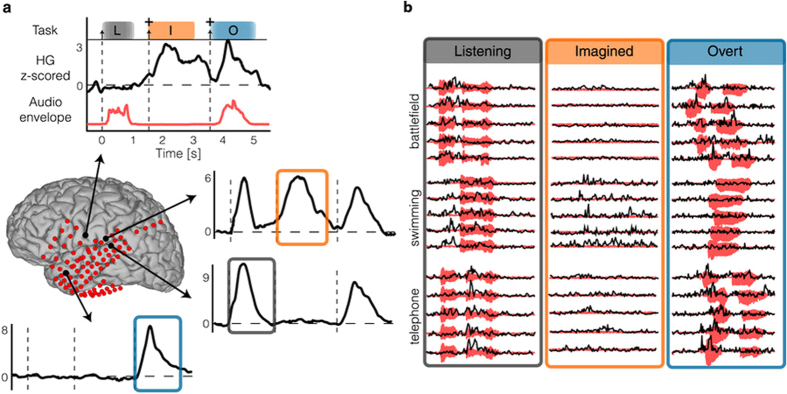


**Figure 3:**