# Supplementary material for: Word pair classification during imagined speech using direct brain recordings
Source: Sci Rep. 2016 May 11;6:25803. doi: 10.1038/srep25803 (PMC4863149; doi:10.1038/srep25803)
Supplement: Supplementary Information [file srep25803-s1.pdf]

Supplementary information

Word pair classification during imagined speech using direct brain recordings

Stephanie Martin[1,2], Peter Brunner[3,4], Iñaki Iturrate[1], José del R. Millán[1], Gerwin Schalk[3,4], Robert T. Knight[2,5], Brian N. Pasley[2]

[1] Defitech Chair in Brain-Machine Interface, Center for Neuroprosthetics, Ecole Polytechnique Fédérale de Lausanne, Switzerland

[2] Helen Wills Neuroscience Institute, University of California, Berkeley, CA, USA

[3] National Center for Adaptive Neurotechnologies, Wadsworth Center, New York State Department of Health, Albany, NY, USA

[4] Department of Neurology, Albany Medical College, Albany, NY, USA

[5] Department of Psychology, University of California, Berkeley, CA, USA

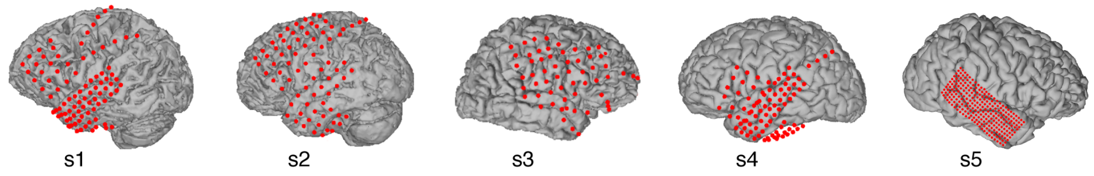

Supplementary Figure S1. Electrode locations. Grid locations for each subject were overlaid on cortical surface reconstructions of each subject's MRI scan.

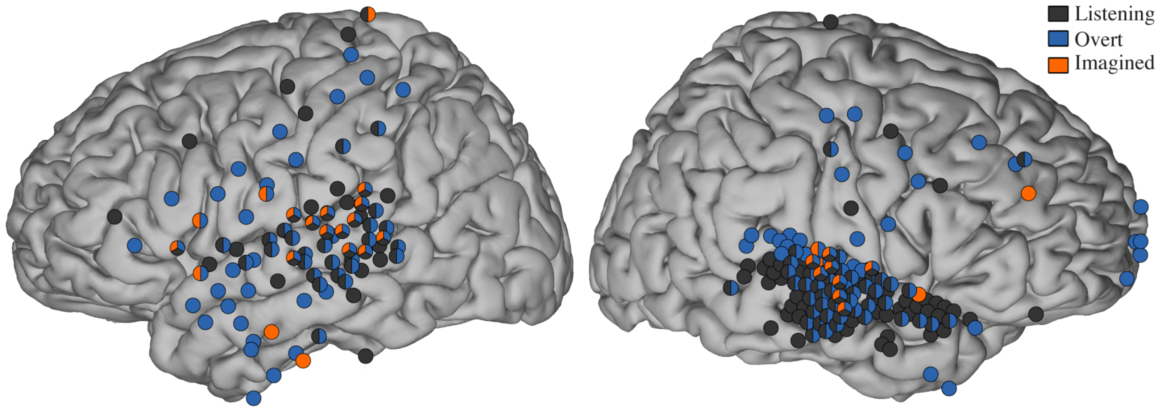

Supplementary Figure S2. Active electrodes across all subjects. We computed the average amplitude from the time course described in Fig. 2a for all three conditions. We computed the coefficient of determination (r^2) between baseline and active state (listening, overt and imagined speech) for each electrodes[49]. To define the significance level of each electrode, we shuffled the labels 1,000 times, and computed r^2. The proportion of shuffled r^2 greater than the observed r^2 yields the p-value that the observed activation is due to chance. Electrodes with $p<0.05$ (corrected for multiple comparison with False Discovery Rate) were plotted on the Talairach brain.

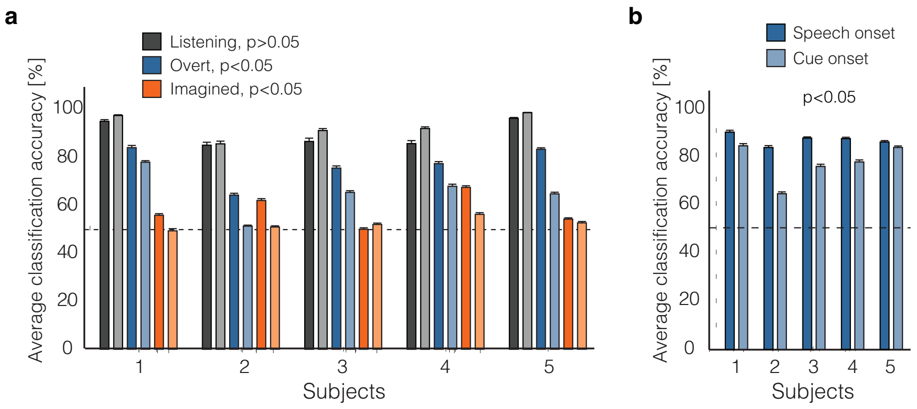

Supplementary Figure S3. Effect of dynamic time warping realignment. a) Average classification accuracy across all pairs of words for each subject and condition (listening, overt and imagined speech) – using DTW (dark) and without DTW (light). Error bars denote resampling SEM. **b)** Comparison of the classification accuracy in the overt speech condition – when epochs were extracted at speech onset or at cue onset. Error bars denote SEM.
